# Trends of insecticide resistance monitoring in mainland Tanzania, 2004–2020

**DOI:** 10.1186/s12936-023-04508-3

**Published:** 2023-03-17

**Authors:** Patrick Tungu, Bilali Kabula, Theresia Nkya, Pendael Machafuko, Edward Sambu, Bernard Batengana, Wema Sudi, Yahaya A. Derua, Victor Mwingira, Denis Masue, Robert Malima, Chonge Kitojo, Naomi Serbantez, Erik J. Reaves, Charles Mwalimu, Samwel L. Nhiga, Mohamed Ally, Humphrey R. Mkali, Joseph J. Joseph, Adeline Chan, Jeremiah Ngondi, Shabbir Lalji, Ssanyu Nyinondi, Erin Eckert, Richard Reithinger, Stephen Magesa, William N. Kisinza

**Affiliations:** 1 National Institute for Medical Research, Amani Medical Research Centre, Muheza, Tanzania; 2 USAID-Okoa Maisha Dhibiti Malaria Project, RTI International, Dar es Salaam, Tanzania; 3 University of Dar es Salaam, Dar es Salaam, Tanzania; 4 U.S. President’s Malaria Initiative, U.S. Agency for International Development, Dar es Salaam, Tanzania; 5 U.S. President’s Malaria Initiative, U.S. Centers for Disease Control and Prevention, Dar es Salaam, Tanzania; 6 National Malaria Control Programme, Ministry of Health, Community Development, Gender, Elderly and Children, Dodoma, Tanzania; 7 U.S. President’s Malaria Initiative, U.S. Centers for Disease Control and Prevention, Atlanta, USA; 8 RTI International, Washington, DC USA

**Keywords:** Insecticide resistance, Malaria vectors, Resistance trends, Tanzania

## Abstract

**Background:**

Insecticide resistance is a serious threat to the continued effectiveness of insecticide-based malaria vector control measures, such as long-lasting insecticidal nets (LLINs) and indoor residual spraying (IRS). This paper describes trends and dynamics of insecticide resistance and its underlying mechanisms from annual resistance monitoring surveys on *Anopheles gambiae *sensu lato (s.l.) populations conducted across mainland Tanzania from 2004 to 2020.

**Methods:**

The World Health Organization (WHO) standard protocols were used to assess susceptibility of the wild female *An. gambiae *s.l. mosquitoes to insecticides, with mosquitoes exposed to diagnostic concentrations of permethrin, deltamethrin, lambdacyhalothrin, bendiocarb, and pirimiphos-methyl. WHO test papers at 5× and 10× the diagnostic concentrations were used to assess the intensity of resistance to pyrethroids; synergist tests using piperonyl butoxide (PBO) were carried out in sites where mosquitoes were found to be resistant to pyrethroids. To estimate insecticide resistance trends from 2004 to 2020, percentage mortalities from each site and time point were aggregated and regression analysis of mortality versus the Julian dates of bioassays was performed.

**Results:**

Percentage of sites with pyrethroid resistance increased from 0% in 2004 to more than 80% in the 2020, suggesting resistance has been spreading geographically. Results indicate a strong negative association (p = 0.0001) between pyrethroids susceptibility status and survey year. The regression model shows that by 2020 over 40% of *An. gambiae* mosquitoes survived exposure to pyrethroids at their respective diagnostic doses. A decreasing trend of *An. gambiae* susceptibility to bendiocarb was observed over time, but this was not statistically significant (p = 0.8413). *Anopheles gambiae* exhibited high level of susceptibility to the pirimiphos-methyl in sampled sites.

**Conclusions:**

*Anopheles gambiae* Tanzania’s major malaria vector, is now resistant to pyrethroids across the country with resistance increasing in prevalence and intensity and has been spreading geographically. This calls for urgent action for efficient malaria vector control tools to sustain the gains obtained in malaria control. Strengthening insecticide resistance monitoring is important for its management through evidence generation for effective malaria vector control decision.

**Supplementary Information:**

The online version contains supplementary material available at 10.1186/s12936-023-04508-3.

## Background

Long-lasting insecticidal nets (LLINs) and indoor residual spraying (IRS) have significantly contributed to the reduction of malaria globally, and remain important interventions for countries to progress towards malaria elimination [1, 2]. In order to achieve 80% nationwide LLINs coverage of at-risk populations in Tanzania, LLINs have been provided since 2009 through health facilities to pregnant women attending antenatal care and children receiving their first measles vaccine [3], to school-age children under the School Net Programme (SNP), under-five catch-up campaigns (U5CC) and universal coverage campaigns (UCC) [4]; the U5CC and UCC alone cumulatively distributed over 60 million LLINs between 2009 and 2020 [5]. As a result, the coverage of LLINs increased nationally from 38.0% in 2008 to 91.5% in 2012 [6]; in 2017, coverage was 77.9% [7].

IRS was introduced in 2007 in Muleba and Karagwe districts in northwest Tanzania [8], with subsequent gradual scale-up across the Lake Victoria regions, covering approximately 6.5 million people in 18 districts by 2012. In 2020 targeted IRS was conducted in six districts with the highest malaria burden in the Lake Victoria regions. These districts were also covered by LLINs.

The scaling-up of LLIN and IRS together with the provision of prompt malaria diagnosis and effective treatment has resulted in the reduction of malaria prevalence in Tanzania, from 14% in 2016 to 7.5% in 2017 for children aged 6–59 months [7]. The effectiveness of these malaria vector control interventions relies on a limited number of insecticides available for public health use and the continued susceptibility of *Anopheles* vectors to these insecticides. Unfortunately, across sub-Saharan Africa and indeed Tanzania, *Anopheles* mosquitoes have been developing resistance to all the classes of available insecticides used for their control [9–11]. The presence of insecticide resistance among malaria vectors presents a challenge to the effectiveness and long-term implementation of insecticide-based vector control interventions in malaria-endemic countries.

Metabolic and target site resistance are the two broad mechanisms by which mosquitoes develop resistance to insecticides. With metabolic resistance, mosquitoes produce increased quantities of enzymes, which either metabolize or sequester the insecticide molecules before reaching the target sites hence rendering them ineffective. A common mosquito insecticide resistance, particularly against pyrethroids and DDT, is caused by non-synonymous mutations to the knockdown resistance (*kdr*) gene, which is conferred by the alteration in the target site of action, i.e., the voltage-gated sodium channel. This genetic resistance mechanism has been widely studied in various insect species and linked to phenotypic resistance [12–14].

To safeguard the efficacy of insecticide-based vector control interventions, evidence-based strategies for preventing and managing insecticide resistance are needed. As per the Global Plan for Insecticide Resistance Management (GPIRM), the WHO urges all malaria endemic countries to develop strategies for monitoring and managing insecticide resistance for the purpose of ensuring that the limited number of insecticides available for vector control remain effective [15].

The National Institute for Medical Research (NIMR) in collaboration with the National Malaria Control Programme (NMCP) and other stakeholder began monitoring insecticide resistance in 1999 with annual monitoring surveys conducted at sentinel sites in more than 15 regions of mainland Tanzania. Formal insecticide resistance monitoring and management strategy has been implemented in the country in 2016 [16]. The strategy provides guidelines on monitoring the susceptibility of malaria vectors to insecticides and how to manage resistance.

While longitudinal *Anopheles* insecticide resistance data is available for Tanzania, these data have never been comprehensively analysed in full, a missed opportunity to offer important information to guide decisions on future malaria vector control efforts in the country.

The aim of this analysis was to describe the evolution and dynamics of insecticide resistance in Tanzania using available results from surveys conducted in surveillance sentinel sites across mainland Tanzania on *Anopheles* mosquito population susceptibility to insecticides and resistance mechanisms to guide future vector control interventions.

## Methods

### Study sites and design

**Fig. 1:**
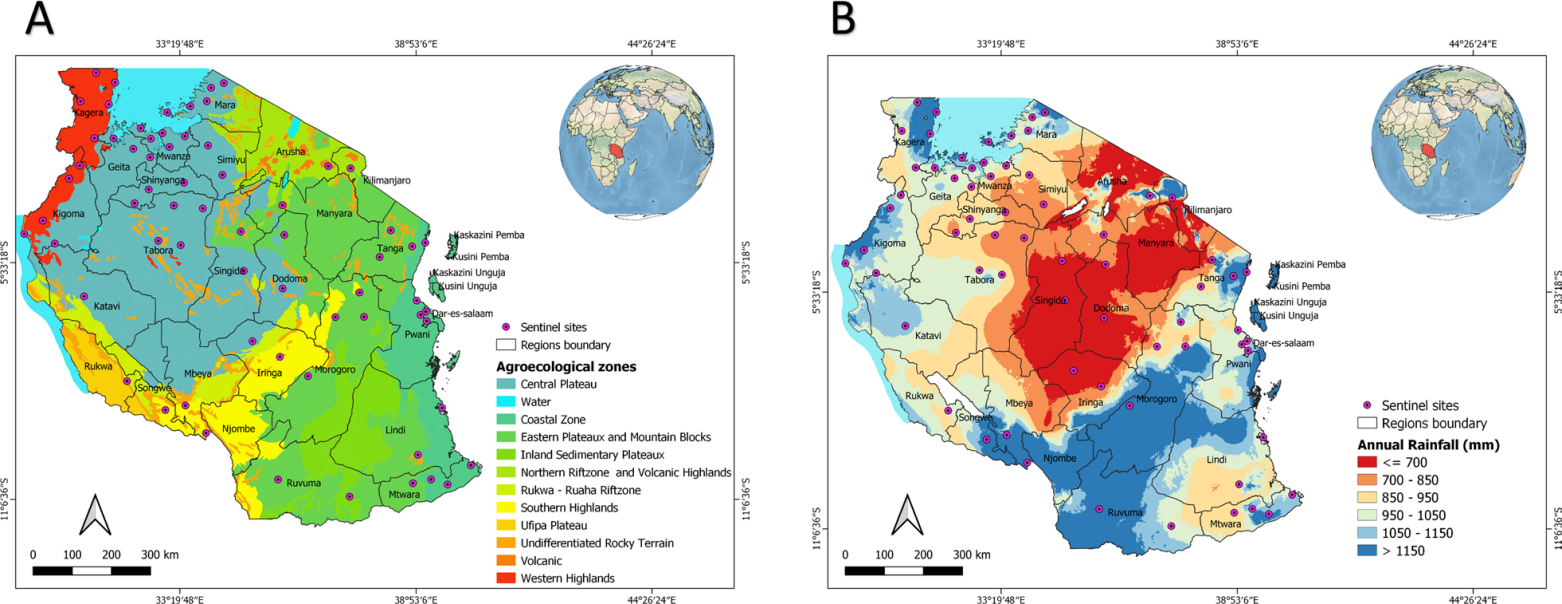
Maps of Tanzania showing the distribution of sentinel sites used for insecticide resistance monitoring by agro-ecological zone (**a**) and annual rainfall (**b**) between 2004 and 2020

This was an analysis of data generated from cross-sectional surveys conducted in selected sentinel surveillance sites to detect and monitor insecticide resistance in *Anopheles* mosquitoes (Fig. 1a and b). Data sources from published and un-published reports are summarized in Table 1. Selection of these sentinel sites was based on WHO recommended criteria, which included: a history of insecticide use in the sampled areas; malaria endemicity; high coverage of vector control interventions, i.e. LLINs and IRS; demographic setting (urban/rural); and site accessibility. The characteristics of the sentinel sites have been previously described by Kabula et al. [9, 17, 18] and in unpublished survey reports (Table 1). Nine sentinel sites were established in 1999 with funding from the Ministry of Health Gender and Elderly and Children (MoHGEC). As LLIN distribution was scaled up, and in response to the concerns around insecticide resistance within the global malaria community, the number of sentinel sites was increased to 11 in 2004, to 14 in 2012, and then to 22 in 2015. Due to changes in malaria epidemiology and geographic targeting of IRS operations, different sites have been sampled over the years, with 71 total sentinel districts having been surveyed across all of the country’s six different agro-ecological zones at one point between 2004 and 2020 (Additional file 1: Table S1). Data prior to 2004 were limited and, therefore, excluded from the analysis.

**Table 1:** Data sources from published and unpublished reports by survey year

Year	Type of data generated	References
2004	Vector species identification and insecticide susceptibility	[9]
2009	Vector species identification and insecticide susceptibility	[9]
2011	Vector species identification and insecticide susceptibility	[9, 18]
2012	Vector species identification, insecticide susceptibility and *kdr* results	[9]
2013–2014	Vector species identification, insecticide susceptibility and *kdr* results	Annual NIMR technical report
2015	Vector species identification, insecticide susceptibility and *kdr* results	[10]
2016–2017	Vector species identification, insecticide susceptibility and *kdr* results	Annual NIMR technical report
2018–2020	Vector species identification, insecticide susceptibility, PBO synergy test and *kdr* results	Annual NIMR technical reports
2004, 2009–2020	Description of the study sites	2004, 2009–2020 Annual NIMR technical reports

### Mosquito collections

Between 2004 and 2008, indoor-resting adult *Anopheles* mosquitoes were collected from human habitations during the rainy season using mechanical aspirators between 0600–0900 h according to a WHO standard protocol for insecticide resistance monitoring [19]. However, because using adult mosquitoes collected through indoor-resting yielded mosquitoes of unknown ages and physiological status which could confound test results, from 2009 onwards *Anopheles* mosquitoes were reared from larvae collected from sentinel sites as described elsewhere [9]. The geographical coordinates of each larvae sampling site were recorded. Larvae collected from several different breeding sites and/or villages in the same district were pooled together for rearing and testing.

### WHO susceptibility bioassays

Two to five-day old female (F1 generation) mosquitoes were tested using standard WHO insecticide susceptibility procedures [19, 20], with 20–25 mosquitoes being placed inside holding tubes before being exposed to WHO test papers treated with either 0.05% deltamethrin, 0.75% permethrin, 0.05% lambdacyhalothrin, 4% DDT, 0.1% bendiocarb or 0.25% pirimiphos-methyl; test papers were prepared at the WHO Collaborating Centre at Universiti Sains, Malaysia [20]. Prior to 2009, insecticide susceptibility tests were conducted to adult mosquitoes collected using indoor resting catches as previously described by Kabula et al. [18].

### Intensity of resistance

Monitoring of insecticide resistance intensity was introduced in 2018. The WHO permethrin and deltamethrin treated papers at 5× and 10× of the discriminating concentrations (DC) were tested to assess the intensity of resistance in 17 out of 22 sentinel sites that recorded resistance to these insecticides. Mosquitoes were first tested to determine their insecticide resistance status in each district (sentinel site), and only those with confirmed resistance were assessed for intensity. Testing procedures and the determination of intensity resistance categories (i.e., low, moderate and high intensity) across locations followed the standard WHO protocol [20]. Briefly, low-intensity resistance means mosquito mortality is < 90% after exposure to 1× DC and ≥ 98% after exposure to 5× DC. Moderate-intensity resistance means mosquito mortality is < 90% after exposure to 1× DC, < 98% after exposure to 5× DC, and is ≥ 98% at 10× DC. High-intensity resistance means mosquito mortality is < 90% after exposure to the 1× DC and < 98% after exposure both to 5× and to 10× DC [9].

### Synergy bioassays

To ascertain involvement of mixed function oxidases in the observed phenotypic resistance, synergy tests using piperonyl butoxide (PBO) were conducted when mosquitoes were found to be resistant to permethrin and/or deltamethrin. Additional bioassays using *An. gambiae* s.l. were done in surveys between 2017 and 2020 using pre-exposure to PBO, a synergist that interferes with the activity of oxidase enzymes that are involved in pyrethroid resistance. Tests were conducted in accordance with standard WHO procedures [20]. Briefly, 2–5 days old F1 adult *An. gambiae* s.l. mosquitoes were pre-exposed to 4% PBO paper for 1 h and immediately exposed to 0.75% permethrin, or 0.05% deltamethrin for another 1 h. Two controls were used during this experiment; the first control constituted mosquitoes exposed to clean papers neither with insecticides nor with PBO, while the second control constituted mosquitoes exposed to papers treated with PBO only [20]. The number of mosquitoes tested for each insecticide varied between 40 and 80. Mortalities recorded 24 h post-exposure were compared between the PBO synergized and the un-synergized (insecticide only) groups. The comparison was used to establish the potential role of cytochrome P_450_ genes in the observed resistance.

### Molecular identification of members of the *Anopheles gambiae* complex

For each sentinel site, DNA samples from 50 randomly selected adult mosquitoes per insecticide per survey were extracted individually. Genomic DNA was extracted according to the method described by Collins et al. [21]. Molecular identification of sibling species of respective *An. gambiae* s.l. were conducted based on PCR methods described by Scott and colleagues [22].

### Detection of knockdown resistance (*kdr*) alleles

Mutations associated with knockdown resistance (*kdr*) L1014S (east mutation) and L1014F (west mutation) in *An. gambiae* s.l. to pyrethroids were assayed using standard TaqMan^®^ quantitative real-time polymerase chain reaction technique (qPCR) [13, 22], using aliquots from the DNA samples extracted for species identification.

### Data analysis

Data from the same sentinel site location district and village and date of sample collection were combined for each species across all bioassays. Percent mortality and 95% confidence intervals for the WHO susceptibility tests were calculated by the binomial exact method using STATA software 13 (Stata Corp LP, College Station, TX, USA). Trend analyses were performed for all tested insecticides. To estimate insecticide resistance trends from 2004 to 2020, aggregated mosquito mortalities from each sentinel site and time were performed using regression analysis of mortality versus the Julian dates of bioassays using STATA software. Only insecticides that were tested across multiple survey years have been included in the analysis. A linear regression model was fitted for species composition as the dependent variable against time (years).

The association between resistance markers and phenotypic resistance was analysed using Chi-square tests on a subsample of *An. gambiae* and *An. arabiensis*. The subsample was weighted by the inverse of the sampling fraction i.e., subsample/total collected to represent the relative proportion in the total population. The *kdr* genotype frequencies among dead and live *An. gambiae* s.l. in susceptibility test across different survey years and clusters were compared using the Genepop software, version 4.0.

### Ethical clearance

Approval was obtained from the ethics committee of the Tanzanian National Institute of Medical Research (Ref: NIMR/HQ/R.8a/VOL.IX/3604). In addition, verbal consent was sought from community leaders of each sentinel site. For mosquito larvae collection, oral consent was obtained from field owners in each location. These locations were not protected land, and the field studies did not involve endangered or protected species.

## Results

### Phenotypic resistance

**Fig. 2:**
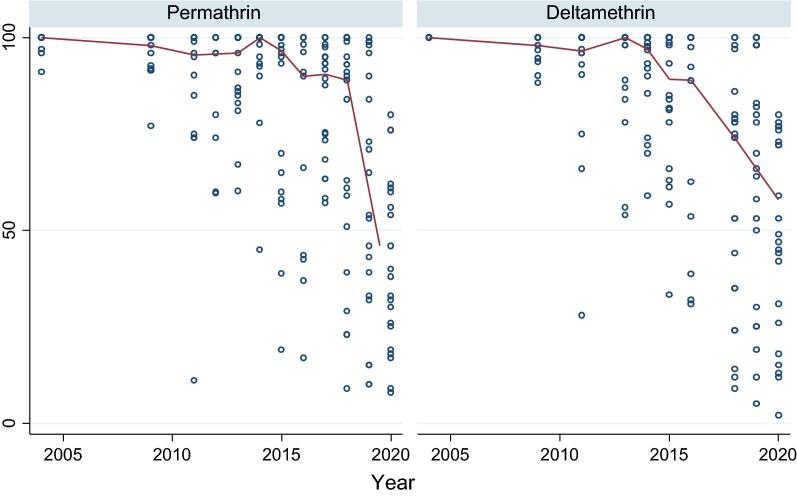
Mortality of *An. gambiae* s.l. exposed to permethrin and deltamethrin over time. Each point denotes the average mortality per site for that year at 95% confidence interval. The line graph shows median value for that year

**Fig. 3:**
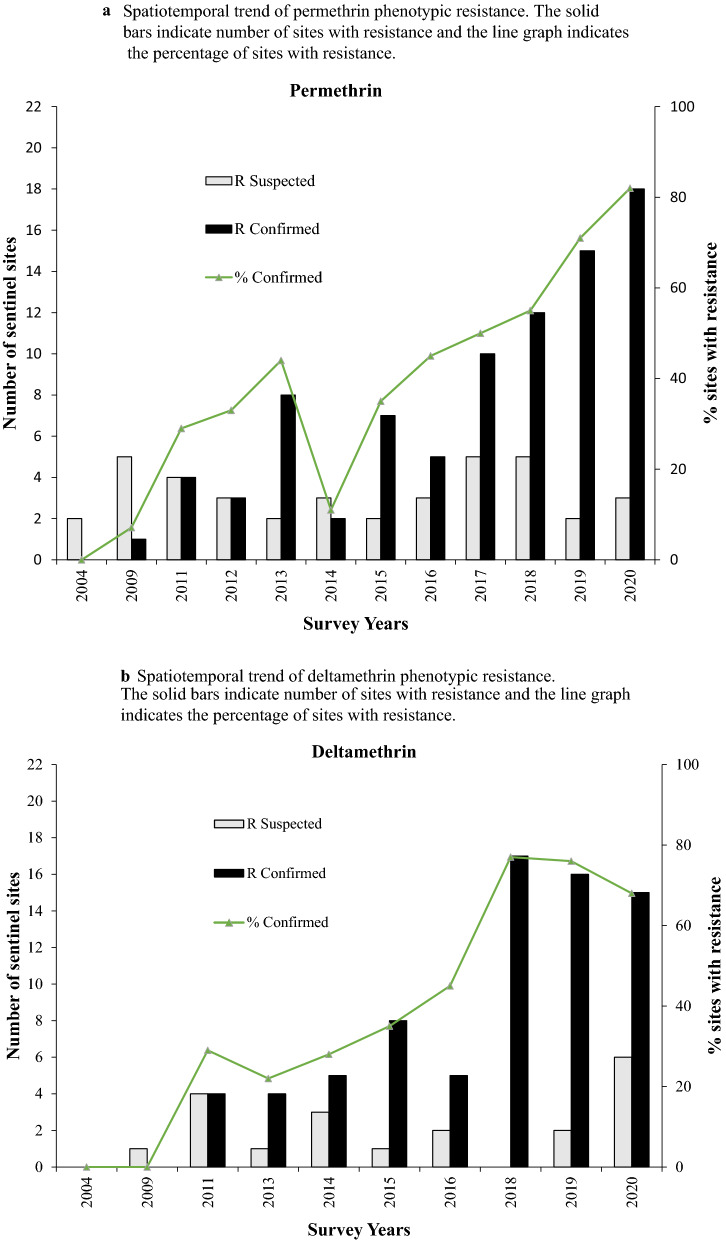
Spatiotemporal trend of insecticide resistance. **a** Spatiotemporal trend of permethrin phenotypic resistance. **b** Spatiotemporal trend of deltamethrin phenotypic resistance. The solid bars indicate number of sites with resistance and the line graph indicates the percentage of sites with resistance

**Fig. 4:**
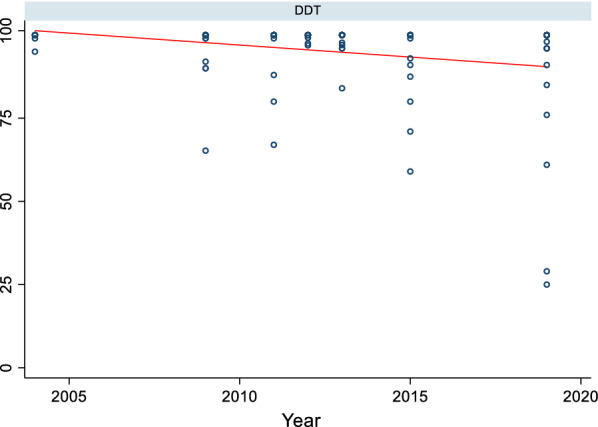
Mortality of *An. gambiae* s.l. exposed to DDT over time. Each point denotes the mortality for a single population with 95% confidence interval. The line is the linear regression best fitting line

**Fig. 5:**
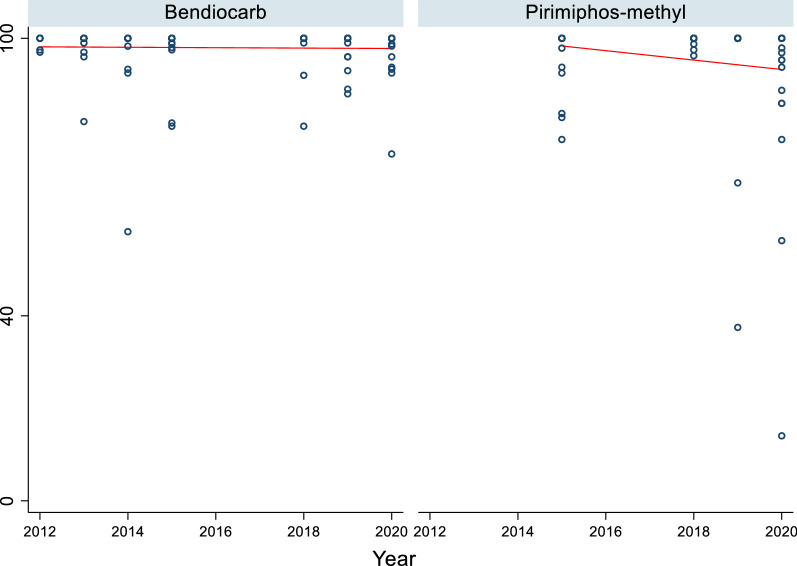
Mortality of *An. gambiae* s.l. exposed to bendiocarb and pirimiphos-methyl over time. Each point denotes the mean mortality for a population in particular a sentinel site with 95% confidence interval. The line is the linear regression best fitting line

**Fig. 6:**
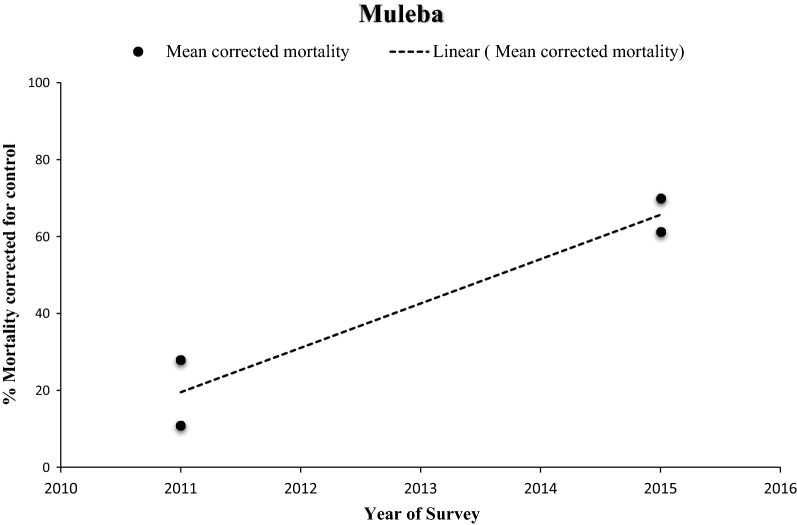
Mortality of *An. gambiae* s.l. from Muleba district exposed to bendiocarb 3 years after it was rotated to pirimiphos-methyl

In 2004, vectors from all surveyed sentinel districts were fully susceptible (100% mortality) to permethrin and deltamethrin (pyrethroids). Thereafter, resistance was reported over time and space. Considering each sentinel site and survey year (time) as a single data point. there has been a statistically significant negative correlation between mortality and time (p = 0.0033) (Fig. 2). Regression analysis showed that by the year 2020 over 45% and 60% of *An. gambiae* s.l. mosquitoes survived exposure to permethrin and deltamethrin. In 2017 *An. gambiae* s.l. from 10 of the 22 sentinel sites were resistant to deltamethrin i.e., Mortality < 90% (Fig. 3a and b). *Anopheles gambiae* s.l. showed reduced susceptibility to lambda-cyhalothrin (another pyrethroid insecticide) over time with a significant negative correlation between mortality and time (p = 0.033). The proportion of sentinel sites with evidence of resistance increased from zero in 2004 to more than 80% in 2020 (Fig. 3a and b). Generally, results indicate a strong negative association (p < 0.0001) between pyrethroids susceptibility and survey year. The estimated odds of pyrethroid susceptibility were significantly less from one survey year to the next. *An. gambiae* s.l. showed decreasing susceptibility levels to DDT over time and there was a negative correlation between DDT mortality and time; however, this was not significant (p = 0.4341) (Fig. 4). Although there was considerable variation among sites and years in the mortality of *An. gambiae* s.l. exposed to bendiocarb (Fig. 5), the decreasing trend of susceptibility over time was not statistically significant (p = 0.8413). However, between 2012 and 2014, when bendiocarb was replaced with an alternative insecticide i.e., pirimiphos-methyl in areas where IRS was being implemented susceptibility to bendiocarb increased significantly but never achieved full susceptibility (F = 23.4, p = 0.04) (Fig. 6). *Anopheles gambiae* s.l. exhibited high levels of susceptibility to pirimiphos-methyl at all sites tested over time (Fig. 5).

### Intensity of resistance

**Fig. 7:**
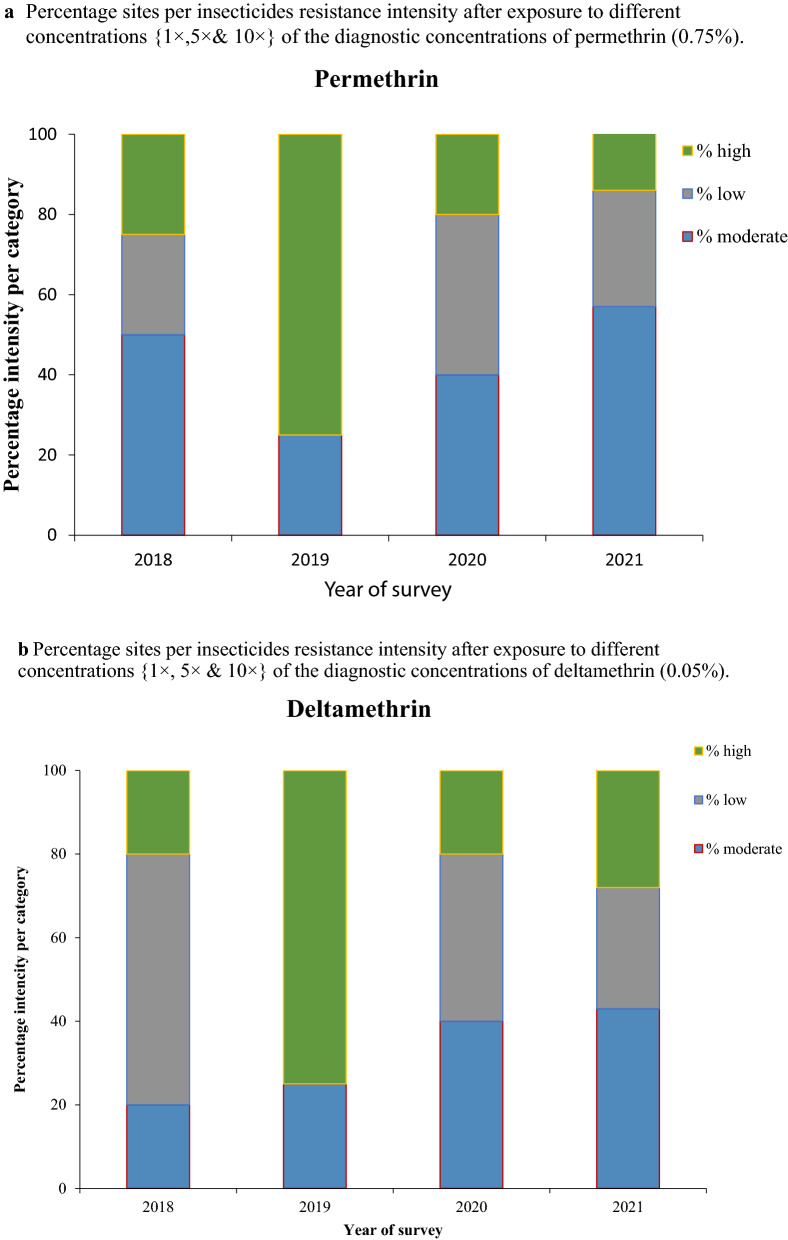
Percentage sites per insecticides resistance intensity after exposure to different concentrations {1×, 5× and 10×} of insecticide. **a** Concentrations of permethrin (0.75%). **b** Concentrations of deltamethrin (0.05%)

Overall, *An. gambiae* s.l. reared from eight, six, and three of the 17 sentinel sites had high, moderate, and low intensity resistance to permethrin, respectively (Fig. 7a). Similarly, *An. gambiae* s.l. reared from seven, five, and five of the 17 sentinel sites had high, moderate and low intensity resistance to deltamethrin (Fig. 7b).

### Species identification

**Fig. 8:**
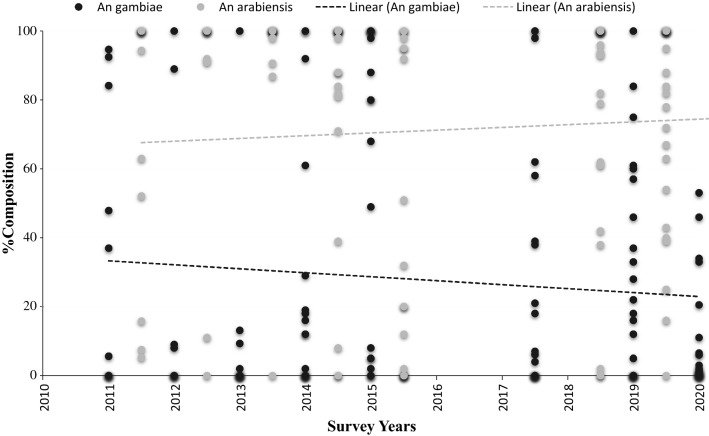
Species composition of *An. gambiae* s.s. and *An. arabiensis* over time. Each point denotes a percentage composition for that survey year. The dashed line is the linear regression best fitting line

Out of *An*. *gambiae* s.l. samples analysed annually between 2010 and 2020, *An*. *arabiensis* represented 71.7% (95% CI 64.6–77.4) and *An*. *gambiae* sensu stricto (s.s.) represented 28.3% (95% CI 22–34.6%). There was a significant positive association over time with *An. arabiensis* (F = 6.7, p < 0.001) and negative non-significant association with *An. gambiae* s.s. (Fig. 8).

### Knockdown resistance (kdr) genotyping

In 2012, the *kdr* east-mutation (L1014S) was detected in *An. gambiae* s.s. in two sentinel sites at allelic frequencies of 20.8% (Geita District) and 88.9% (Ngara District). In 2013, the *kdr* east-mutation was detected in *An. arabiensis* at one sentinel site (Kinondoni District) at an allelic frequency of 17.0%. In 2014, the *kdr* east-mutation was detected in *An. arabiensis* from sentinel sites in Muheza, Kinondoni, Musoma, Ngara and Kyela Districts at allelic frequencies ranging from 6.0 to 25.0%. In 2015, *kdr* east-mutation was detected in *An. gambiae* s.s. in Muleba District at a frequency of 0.6%. In 2016, the *kdr* east-mutation was detected in *An. gambiae* s.s. in two sentinel sites with allelic frequencies of 83.0% (Muleba District) and 53.0% (Ruangwa District). In 2012, *kdr* west-mutation (L1014F) was detected in both *An. gambiae* s.s. and *An. arabiensis* in Muleba District at allelic frequencies of 100% and in *An. arabiensis* in Arumeru District at an allelic frequency of 0.6%. In 2019, *kdr* east-mutation was detected in *An. gambiae* s.s. mosquitoes in 9 (41%) of the 22 sampled sentinel sites, with allelic frequencies ranging from 3.0% in Kilwa District to 93.0% in Misenyi District (Table 2). In 2019, *kdr* east-mutation was detected in *An. arabiensis* mosquitoes in 11 (50%) of the 22 sampled sentinel sites, with allelic frequencies ranging from 2.0% in Kilwa and Geita Districts to 63.0% in Tandahimba District (Table 2). In 2020, *kdr* east-mutation was also detected in *An. gambiae* s.s. mosquitoes with allelic frequencies ranging from 15.0% in Bukoba District to 50.0% in Kigoma District Council (Table 3). The *kdr* east-mutation was detected in *An. arabiensis* with allelic frequencies ranging from 1.0% in Ngara and Ruangwa Districts to 8.0% in Misenyi District (Table 4).

**Table 2:** Distribution and allelic frequencies of L1014S (*kdr* east-mutation genotypes) in 2019

Sentinel District	*An. gambiae* s.s					*An. arabiensis*				
		Genotype count			Allelic frequency		Genotype count			Allelic frequency
	N	RR	RS	SS	R	N	RR	RS	SS	R
Kilosa	33	0	0	33	0.00	41	0	8	33	0.12
Geita	0	0	0	0	0.00	72	1	1	70	0.02
Kilwa	48	0	3	45	0.03	22	0	4	18	0.09
Kakonko	0	0	0	0	0.00	67	3	3	61	0.07
Ukerewe	67	40	8	19	0.66	0	0	0	0	0.00
Newala	0	0	0	0	0.00	67	2	12	53	0.12
Meatu	67	4	3	60	0.08	0	0	0	0	0.00
Tandahimba	0	0	0	0	0.00	67	26	32	9	0.63
Kasulu	25	0	4	21	0.08	42	0	0	42	0.00
Tunduru	19	0	16	3	0.40	49	0	0	49	0.12
Kibondo	56	49	3	4	0.90	11	4	4	3	0.55
Misenyi	46	40	1	5	0.93	27	5	2	20	0.22
Nachingwea	3	0	1	2	0.17	64	0	2	62	0.02
Biharamuro	22	20	0	2	0.91	45	11	0	34	0.24

*RR* homozygous resistant, *RS* heterozygous resistant, *SS* homozygous susceptible

**Table 3:** Distribution and allelic frequencies of *kdr* mutations in *Anopheles gambiae* s.s. among the six sentinel districts registered with *kdr* resistance in 2020

Sentinel District	N	Genotype count for *Anopheles gambiae* s.s							
		*kdr*-east (L1014S)			Allelic frequency	*kdr*-west (L1014F)			Allelic frequency
		RR	RS	SS	R	RR	RS	SS	R
Bukoba DC	90	13	2	65	0.15	0	0	90	0.00
Kakonko	6	0	0	6	0.00	0	2	4	0.17
Kigoma DC	4	2	0	2	0.50	0	0	4	0.00
Misenyi	104	49	0	55	0.47	0	0	104	0.00
Ngara	40	10	0	30	0.25	0	0	40	0
Ruangwa	12	0	0	12	0.00	0	0	12	0

*RR* homozygous resistant, *RS* heterozygous resistant, *SS* homozygous susceptible

**Table 4:** Distribution and allelic frequencies of *kdr* mutations in *Anopheles arabiensis* among the six sentinel districts registered with *kdr* resistance in 2020

Sentinel District	N	Genotype count for *Anopheles arabiensis*							
		*kdr*-east (L1014S)			Allelic frequency	*kdr*-west (L1014F)			Allelic frequency
		RR	RS	SS	R	RR	RS	SS	R
Bukoba DC	110	8	1	101	0.80	0	0	110	0.00
Kakonko	194	0	6	188	0.01	0	11	183	0.03
Kigoma DC	196	4	7	175	0.04	0	4	192	0.01
Misenyi	96	8	0	88	0.08	0	0	96	0.00
Ngara	160	1	0	159	0.01	1	0	159	0.01
Ruangwa	188	2	0	186	0.005	0	0	188	0.00

*RR* homozygous resistant, *RS* heterozygous resistant, *SS* homozygous susceptible

### Synergy bioassays

**Fig. 9:**
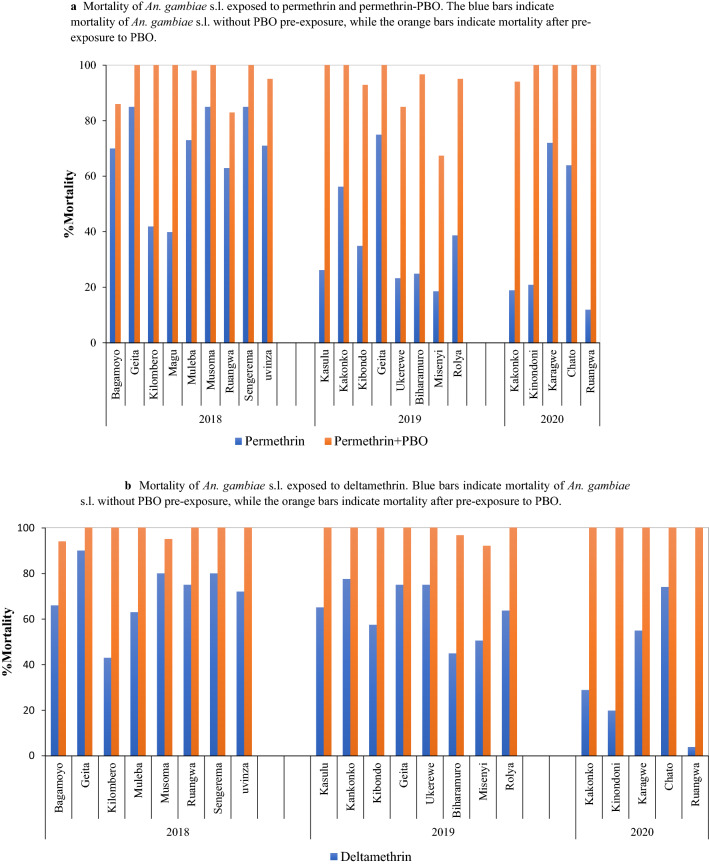
Mortality of *An. gambiae* s.l. exposed to insecticides. **a** Mortality of *An. gambiae* s.l. exposed to permethrin and permethrin-PBO. **b** Mortality of *An. gambiae* s.l. exposed to deltamethrin. The blue bars indicate mortality of *An. gambiae* s.l. without PBO pre-exposure, while the orange bars indicate mortality after pre-exposure to PBO

The effect of the PBO synergist was evident in sentinel sites where synergy tests were carried out with mortality rate range 85–100% for the permethrin and deltamethrin, confirming involvement of monooxygenase enzyme activity in the pyrethroid detoxification. For permethrin alone, mortality ranged from 12 to 85% (Fig. 9a). At the same sites, mortality to permethrin after exposure to PBO ranged from 67.5 to 100% (Fig. 9a). For deltamethrin alone, mortality in mosquitoes ranged from 4 to 90%. After exposure to PBO, mortality among *An. gambiae* s.l. exposed to deltamethrin ranged from 92 to 100% (Fig. 9b).

## Discussion

Between 2009 and 2020, insecticide resistance in *An. gambiae* s.l. in Tanzania was not only increasing in frequency, it was as well spreading geographically. However, results from entomological surveys conducted between 2004 and 2009 demonstrate that *An. gambiae* s.l. populations were susceptible to all tested insecticides, including pyrethroids. Nevertheless, the West African leucine-phenylalanine (L1014F *kdr*) mutations wad detected in this time period in one sentinel site [23]. In 2010 and 2011, pyrethroid resistance was detected in *An. gambiae* s.l. in 34% of all the sites surveyed. By 2014, resistance to permethrin and deltamethrin was detected in *An. gambiae* s.l. in 64% and 70% of sampled sentinel sites, respectively. By 2020, resistance to permethrin, deltamethrin, and lambda-cyhalothrin was detected in over 90% of sampled sentinel sites. In nearly one quarter of the sentinel sites in 2020, the observed pyrethroid resistance was of high intensity, meaning that mosquitoes were able to survive the exposure to insecticides at both 5× and 10× DC. Results from these studies also show that both target site voltage gated sodium channel (vgsc) and metabolic mechanisms are associated with the reported resistance. The target sites *kdr* east-mutation-(L1014S) and *kdr* west-mutations (L1014F) associated with DDT and pyrethroids were detected within the *An. gambiae* s.l. populations. Furthermore, synergist test results indicate that metabolic resistance is partially or fully involved in the observed phenotypic resistance. This indicates that controlling mosquitoes using pyrethroid-based vector control interventions without factoring in a resistance management plan will be difficult.

Results of the analyses presented here confirm what was earlier reported in a series of previous studies in Tanzania. The first published report of insecticide resistance in wild *An. gambiae* s.l*.* was recorded against permethrin in Moshi in 2006, with West African leucine-phenylalanine (L1014F *kdr*) mutations being identified [23], followed by a report of permethrin resistance in the same area due to elevated levels of both mixed function oxidases and β-esterases [24]. Furthermore, between 2009 and 2010, resistance to DDT was observed in Ilala District in Dar es Salaam, and resistance to lambdacyhalothrin and permethrin were observed in Moshi District [9]. Resistance to other pyrethroids (permethrin and deltamethrin) was also observed in Muleba District, with bioassay mortality of anopheline mosquitoes exposed to these pyrethroids consistently less than 35% [25]. There was a sharp decrease in mortality of *An.* *gambiae* s.l. exposed to bendiocarb after two rounds of bendiocarb‐based IRS in Muleba district [26]. *An. gambiae* s.l. from other Districts was reported to have developed insecticide resistance to pyrethroids, DDT, and bendiocarb [10].

The rapid increase in pyrethroid resistance across Tanzania is most likely attributed to the cumulative effects of increased use of insecticide-treated nets, that began in the 1990s, followed by national scaling up of mass distribution of LLINs to achieve universal coverage in 2011 and consistently maintained to date. The contribution of LLINs in the development of pyrethroid resistance in malaria vectors has been reported throughout Africa [27–29]. In Tanzania, coverage of LLINs has been scaled-up in the country since 2011 when the initial 8.7 million LLINs were distributed [30]. Most of the LLINs that have been distributed since that time and are in use are treated with permethrin [7] or deltamethrin. The presence of millions of pyrethroid-treated LLINs in the field presents selection pressure for pyrethroids resistance.

IRS operations, which previously have been implicated in the selection of pyrethroid resistance [31], also may have compounded selection pressure in locations where IRS with lambdacyhalothrin was applied, such as Muleba District. Since being launched in 2007 in Muleba and Karagwe Districts, IRS was expanded in 2009 to cover up to 18 districts of Kagera, Mwanza, and Mara Region located around Lake Victoria. From 2012 onwards, IRS discontinued use of pyrethroids and only has been applied in a few targeted districts in the same Lake Victoria zone. Though not part of the NMCP’s strategy for malaria control, the frequent use of aerosol spray and coils, some of which include pyrethroids, in urban areas could have contributed to selective pyrethroid pressure in mosquito populations. Lastly, extensive use of pyrethroids in agriculture might also have contributed to the observed insecticide resistance pattern.

Given that carbamates are used for veterinary and public health purposes, the observed resistance to this class of insecticides in *An. gambiae* s.l. is not surprising. It is possible that the observed carbamate resistance in the selected areas is due to the use of carbamates for IRS and other insecticides as herbicides in agriculture, as reported in previous studies showing that the use of this product contributes to resistance [32–34]. The rise of carbamate resistance has precluded the continued use of bendiocarb for IRS in Tanzania, although use of this insecticide in neighboring Mozambique seemed to be effective despite high levels of bendiocarb resistance observed there [35].

Despite high levels of resistance to pyrethroids and carbamates, *An. gambiae* s.l. populations have remained susceptible to pirimiphos-methyl (organophosphate) in all sentinel sites across the country between 2015 and 2020. This may be due to the fact that organophosphates are less widely used compared to pyrethroids. Currently, pirimiphos-methyl is used for IRS in six districts only. Similar results have been reported in Malawi [36]*.* Earlier results obtained from Dar es Salaam showed unexpected high resistance of *An. gambiae* to DDT [9], and it was speculated that there could be selection due to cross-resistance associated with pyrethroids since both share the same mode of action [37].

The development of pyrethroid resistance has important implications for malaria vector control [38–40]. Pyrethroids have been used for both LLINs and IRS and loss of effectiveness against mosquitoes has serious consequences. The development of pyrethroid resistance in *An. funestus* in South Africa resulted in a sharp increase in malaria cases and consequently necessitated a change to DDT in combination with pyrethroids for IRS [41, 42]. Although IRS may be implemented with several non-pyrethroid insecticides, most of these are significantly more expensive than pyrethroids. As control programmes have been switching to non-pyrethroids as pyrethroid resistance expanded, the programmatic coverage of IRS decreased and, in some cases, NMCPs abandoned IRS altogether. Unfortunately, most available LLINs are treated with pyrethroids and resistance is a serious concern given that it has been estimated that 68% of the malaria cases averted since 2000 are attributable to LLINs [1]. There are several previous studies that shows pyrethroid resistance is undermining the effectiveness of LLINs [38–40, 43]. Laboratory experiments in which wild, resistant *Anopheles* strains directly exposed to net samples have indicated reduced efficacy of LLINs against resistant strains, while both experimental hut studies and field studies have demonstrated that resistant mosquitoes are more likely to enter LLINs, feed upon the occupants, and survive [44, 45]. A case–control study carried out in Malawi showed that among sick children aged < 5 years old reporting to the main referral hospital in the area, there was no protective effect of LLIN use [31]. The observed high pyrethroid resistance intensity might have epidemiological significance by compromising the effectiveness of LLINs in the community, and resulting in LLIN programmatic failure [46].

The spread of insecticide resistance in Tanzania described in this analysis suggests a need for insecticide resistance management for malaria vector control. WHO GPIRM provides recommendations for managing resistance in malaria endemic countries that includes the use of alternative insecticides in rotation or combination delivered through LLINs or IRS. Modelling has shown that the most promising way to delay the selection of resistance is to apply mixtures of unrelated insecticides [47]. Rather than use a non-pyrethroid insecticide to overcome resistance, an equally valid approach is to deploy a chemical synergist on the fibers. PBO is one of the synergists that overcomes resistance by inhibiting the enzymes responsible for certain types of resistance, essentially reversing resistance to pyrethroids. In both the laboratory [44, 48] and field [49] *An. gambiae* s.l. mortality was shown to be higher when PBO LLINs are used compared to pyrethroid-only LLINs; this study’s data showed synergy with PBO managed to restore the efficacy of permethrin and deltamethrin in killing mosquitoes in all sentinel sites. Furthermore, cluster randomized trials have shown a 44% reduction in morbidity in the area where PBO LLINs were distributed and used as compared to areas with standard pyrethroid-only LLINs [46]. These results suggest that PBO LLINs [50] may be a potential resistance management option in Tanzania.

Similarly, insecticide rotation for IRS is also a GPIRM recommended option for managing insecticide resistance. Results from our analysis indicate a significant increase in the susceptibility of bendiocarb in Muleba district after 3 years of pirimiphos-methyl use for IRS. The availability of the newly WHO prequalified insecticide products, Sumishield™ (clothianidin, a neonicotinoid) and Fludora Fusion, currently in use for IRS in Tanzania, provides more options that allow national malaria control programmes to implement suitable resistance management strategies. In addition, new insecticides and vector control tools are expected to enter the market in the near future, like new generation nets, such as Interceptor G2 [45], that are expected to add additional effective insecticide resistance management options.

In all surveys and sites, *An. arabiensis* was the predominant species, followed by *An. gambiae* s.s. The role of *An. arabiensis* in outdoor biting, particularly early in the evening hours (before bed time), may result in outdoor residual malaria transmission as described in parts of Tanzania [51]. Ecologically, *An. arabiensis* is known to have a wide range of behaviour patterns, making it more difficult to control compared to *An. gambiae* s.s.

One potential limitation of this analysis is that the mosquitoes used in the susceptibility testing were collected using different methods. Initially from 2004 to 2008, indoor-resting *Anopheles* mosquitoes of unknown ages and physiological status were collected from human habitations and tested for their susceptibility status. From 2009 onwards, *Anopheles* mosquitoes were reared from larvae collected from sentinel sites. In some sentinel sites, larvae were collected from only a few habitats; therefore, resistance results for the larger district area may be misrepresented.

## Conclusion

The major malaria vector in Tanzania, *Anopheles gambiae* s.l. is currently highly resistant to pyrethroids across the country, with increasing prevalence and intensity spreading geographically since 2009. However, the efficacy of permethrin and deltamethrin was partially of fully restored with pre-exposure to PBO in all sentinel districts in which synergy study was carried out, suggesting a significant role of cytochrome P_450_ detoxification enzymes in the observed pyrethroid resistance. Thus, the use of PBO LLINs and other next-generation LLINs, as well as rotation of insecticides with different modes of action for IRS guided by the ongoing insecticide resistance monitoring, might be effective strategies to mitigate resistance and ensure effectiveness of these vital interventions.

## Supplementary Information

**Additional file 1: Table S1.** List of sentinel sites and their respective agro-ecological zones, 2004–2020.

## Data Availability

The datasets used and/or analysed during the current study are available from the corresponding author on reasonable request.
