# Supplementary material for: Trends of insecticide resistance monitoring in mainland Tanzania, 2004–2020
Source: Malar J. 2023 Mar 17;22:100. doi: 10.1186/s12936-023-04508-3 (PMC10024418; doi:10.1186/s12936-023-04508-3)
Supplement: Supplementary file 1 — Additional file 1: Table S1. List of sentinel sites and their respective agro-ecological zones, 2004–2020. [file 12936_2023_4508_MOESM1_ESM.docx]

**Additional Table S1: List of sentinel sites and their respective agro-ecological zones, 2004-2020**

| **Agro-ecological Zone** | **Sentinel Site (District)** | **Region** | **Average Annual Rainfall (mm)** | **Coordinates** |
| --- | --- | --- | --- | --- |
| Central Zone | Babati | Manyara | 700-850 | 4.2078° S, 35.7461° E |
| Central Zone | Dodoma | Dodoma | <700 | 6.1630° S, 35.7516° E |
| Central Zone | Igunga | Tabora | 750-850 | 4.2846° S, 33.8702° E |
| Central Zone | Kondoa | Dodoma | <700 | 4.9068° S, 35.7898° E |
| Central Zone | Manyoni | Singida | <700 | 5.7482° S, 34.8344° E |
| Central Zone | Meatu | Simiyu | 700-850 | 3.4979° S, 34.3310° E |
| Central Zone | Nzega TC | Tabora | 700-850 | 4.2161° S, 33.1858° E |
| Central Zone | Nzega DC | Tabora | 700-850 | 4.2161° S, 33.1858° E |
| Central Zone | Singida | Singida | <700 | 4.8254° S, 34.7630° E |
| Central Zone | Tabora | Tabora | 700-850 | 5.0425° S, 32.8197° E |
| Central Zone | Ushetu | Shinyanga | 700-850 | 4.1610° S, 32.2629° E |
| Central Zone | Uyui | Tabora | 950-1050 | 5.1456° S, 33.3486° E |
| Eastern Zone (Inland) | Kilombero | Morogoro | >1150 | 8.2215° S, 36.3498° E |
| Eastern Zone (Inland) | Kilosa | Mvomero | >1150 | 6.8343° S, 36.9917° E |
| Eastern Zone (Inland) | Muheza | Tanga | 850-950 | 5.1759° S, 38.7918° E |
| Eastern Zone (Inland) | Kibaha | Pwani (Coastal) | 950-1050 | 6.7831° S, 38.9910° E |
| Eastern Zone (Coastal) | Bagamoyo | Pwani (Coastal) | 950-1050 | 6.4456° S, 38.8989° E |
| Eastern Zone (Coastal) | Ilala | Dar es salaam | 1050-1151 | 6.9276° S, 39.1336° E |
| Eastern Zone (Coastal) | Kilwa | Lindi | 850-950 | 8.9659° S, 39.4971° E |
| Eastern Zone (Coastal) | Kinondoni | Dar es salaam | 1050-1150 | 6.7053° S, 39.1127° E |
| Eastern Zone (Coastal) | Mtwara | Mtwara | 1050-1150 | 10.3112° S, 40.1760° E |
| Eastern Zone (Coastal) | Mtwara DC | Mtwara | 1050-1150 | 10.3112° S, 40.1760° E |
| Eastern Zone (Coastal) | Newala | Mtwara | 1050-1150 | 10.6417° S, 39.2376° E |
| Eastern Zone (coastal) | Tandahimba | Mtwara | 1050-1150 | 10.7620° S, 39.6260° E |
| Eastern Zone (coastal) | Tanga | Tanga | >1150 | 5.0889° S, 39.1023° E |
| Eastern Zone (inland) | Handeni | Tanga | 850-950 | 5.4236° S, 38.0261° E |
| Eastern Zone (inland) | Morogoro | Morogoro | >1150 | 6.8278° S, 37.6591° E |
| Eastern Zone (inland) | Mvomero | Mvomero | >1150 | 6.2555° S, 37.5535° E |
| Eastern Zone (inland) | Nachingwea | Lindi | 850-950 | 2.5164° S, 32.9175° E |
| Eastern Zone (inland) | Ruangwa | Lindi | 850-950 | 10.0663° S, 38.9277° E |
| Eastern Zone (inland) | Lushoto | Tanga | 850-950 | 4.7987° S, 38.2902° E |
| Eastern Zone (inland) | Masasi | Mtwara | 850-950 | 10.7324° S, 38.8101° E |
| Lake Zone (central) | Buchosa | Mwanza | 850-950 | 2.2497°S, 32.2427°E |
| Lake Zone (central) | Butiama | Mara | 1050-1150 | 1.7677° S, 33.9645° E |
| Lake Zone (central) | Chato | Geita | 1050-1151 | 2.6382° S, 31.7668° E |
| Lake Zone (central) | Magu | Mwanza | 850-950 | 2.5901° S, 33.4469° E |
| Lake Zone (central) | Misungwi | Mwanza | 950-1050 | 2.8403° S, 33.0851° E |
| Lake Zone (central) | Musoma DC | Mara | 1050-1150 | 1.8858° S, 33.5728° E |
| Lake Zone (central) | Mwanza | Mwanza | 1050-1150 | 2.5164° S, 32.9175° E |
| Lake Zone (central) | Nyang`wale | Geita | 850-950 | 3.0856° S, 32.6277° E |
| Lake Zone (central) | Rorya | Mara | 1050-1150 | 1.4531° S, 34.0641° E |
| Lake Zone (central) | Sengerema | Mwanza | 1050-1150 | 2.6471° S, 32.6447° E |
| Lake Zone (central) | Tarime | Mara | 1050-1150 | 1.3436° S, 34.3643° E |
| Lake Zone (central) | Ukerewe | Mwanza | 1050-1150 | 2.0299° S, 33.0339° E |
| Lake Zone (central) | Bariadi | Simiyu | 700-850 | 2.8070° S, 33.9917° E |
| Lake Zone (central) | Kahama | Shinyanga | 850-950 | 3.8376° S, 32.5938° E |
| Lake Zone (central) | Shinyanga DC | Shinyanga | 700-850 | 3.6810° S, 33.4271° E |
| Lake Zone (central) | Geita | Geita | 850-1050 | 2.8850° S, 32.2314° E |
| Lake Zone (western highlands) | Biharamulo | Kagera | 950-1050 | 2.6343° S, 31.3141° E |
| Lake Zone (western highlands) | Bukoba DC | Kagera | >1150 | 1.3296° S, 31.8050° E |
| Lake Zone (western highlands) | Karagwe | Kagera | 950-1050 | 1.7718° S, 30.9876° E |
| Lake Zone (western highlands) | Misenyi | Kagera | >1150 | 1.1009° S, 31.3542° E |
| Lake Zone (western highlands) | Muleba | Kagera | >1150 | 1.8413° S, 31.6559° E |
| Lake Zone (western highlands) | Ngara | Kagera | 950-1050 | 1.2781° S, 36.8304° E |
| Northern Zone | Arumeru | Arusha | >1150 | 3.2923° S, 36.8250° E |
| Northern Zone | Moshi | Kilimanjaro | >1150 | 3.3430° S, 37.3507° E |
| Southern highlands | Iringa | Iringa | <700 | 7.7681° S, 35.6861° E |
| Southern highlands | Kyela | Mbeya | >1150 | 9.5563° S, 33.9446° E |
| Southern highlands | Mbeya | Mbeya | >1150 | 8.9094° S, 33.4608° E |
| Southern highlands | Mbozi | Songwe | >1150 | 9.0141° S, 32.9888° E |
| Southern highlands | Songea | Ruvuma | >1150 | 10.6463° S, 35.6424° E |
| Southern highlands | Sumbawanga DC | Rukwa | 950-1050 | 8.3368° S, 32.0837° E |
| Southern highlands | Iringa Rural | Iringa | <700 | 7.3995° S, 35.0388° E |
| Southern highlands | Mpanda | Katavi | 1050-1150 | 6.3478° S, 31.0736° E |
| Southern highlands | Nyasa | Ruvuma | 950-1050 | 11.6701° S, 34.6857° E |
| Southern highlands | Tunduru | Ruvuma | 950-1050 | 11.0407° S, 37.3293° E |
| Western highlands | Kakonko | Kigoma | 950-1050 | 3.2799° S, 30.9661° E |
| Western highlands | Kasulu | Kigoma | 1050-1150 | 4.5702° S, 30.1012° E |
| Western highlands | Kibondo | Kigoma | 950-1050 | 3.5838° S, 30.7178° E |
| Western highlands | Kigoma DC | Kigoma | 950-1051 | 4.8824° S, 29.6615° E |
| Western highlands | Uvinza | Kigoma | 1050-1150 | 5.1069° S, 30.3839° E |

*DC=district council; TC=town council*
